# Errata

**Published:** 2015-02-20

## Vol. 64, No. 5

In the report, “Notes from the Field: Prevalence of Risk Factors for Suicide Among Veterinarians — United States, 2014,” an error occurred in the author affiliations. The author list and the author affiliations should read as follows:

Randall J. Nett, MD^1^,^2^, Tracy K. Witte, PhD^3^, Stacy M. Holzbauer, DVM^1^,^4^, Brigid L. Elchos, DVM^5^, Enzo R. Campagnolo, DVM^1^,^6^, Karl J. Musgrave, DVM^7^, Kris K. Carter, DVM^1^,^8^, Katie M. Kurkjian, DVM^1^,^9^, Cole Vanicek, DVM^10^, Daniel R. O’Leary, DVM^1^,^7^, Kerry R. Pride, DVM^2^, Renee H. Funk, DVM^11^

## Vol. 64, No. 4

In the report “Mortality Among Blacks or African Americans with HIV Infection — United States, 2008–2012,” the second sentence on page 84 should read, “The report evaluates all-cause mortality in persons living with HIV and does **not** measure mortality resulting from HIV.”

## Footnotes

1 Division of State and Local Readiness, Office of Public Health Preparedness and Response, CDC;

2 Montana Department of Public Health and Human Services;

3 Department of Psychology, Auburn University;

4 Minnesota Department of Health;

5 Mississippi Board of Animal Health;

6 Pennyslvania Department of Health;

7 Wyoming Department of Health;

8 Idaho Department of Health and Welfare;

9 Virginia Department of Health;

10 Nebraska Department of Health and Human Services;

11 Emergency Preparedness and Response Office, National Institute of Occupational Safety and Health, CDC

